# Supplementary material for: Trimethoprim-Sulfamethoxazole-associated early neutropenia in Mexican adults living with HIV: A cohort study
Source: PLoS One. 2023 May 11;18(5):e0285541. doi: 10.1371/journal.pone.0285541 (PMC10174569; doi:10.1371/journal.pone.0285541)
Supplement: S1 Appendix — (DOCX) [file pone.0285541.s001.docx]

**S1 Appendix. Collected independent variables.**

| Conceptualization | Operationalization | Unit of measure or categories |
| --- | --- | --- |
| Hospital stay | Days of hospitalization at INER | Days |
| HIV viral load  (HIV VL) | Serum HIV viral load during the observation period  (Undetectable will be VL=1, and detectable <40 will be VL=39) | Copies/ml |
| CD4 T-cell count | Serum CD4 T-cells count during the observation period | Cells/mm^3^ |
| Clinical stage of HIV infection | CDC Classification System for HIV Infection in the clinical record | Stage A1, A2, A3, B1, B2, B3, C1, C2 or C3 |
| HIV Risk Group | Registration of the WHO classification in the clinical record | -Sex between men  -Intravenous drug use  -Heterosexual  -Other/ Unknown |
| Time from HIV diagnosis | Time in months from HIV diagnosis to study onset | Months |
| Body mass index (BMI) | Calculation of BMI by weight in kg divided by the square of height in meters (kg/m^2^) and categorization | <18.5 (low weight)  18.5-24.9 (normal weight)  25.0-29.9 (overweight)  ≥30.0 (obesity) |
| Glomerular filtration rate (GFR) | Mean GFR during follow-up by the CKD-EPI formula and categorization | ≥60 ml/min  <60 ml/min |
| Complete blood count (CBC) | Complete blood count test results | Depending on the variable |
| Number of days of CBC testing | Number of days with complete blood count test | Days |
| Comorbidities | Diseases recorded in the patient's clinical record (sepsis, hypersplenism, infections, diabetes, hypertension, heart disease, nephropathy, rheumatological/autoimmune diseases, aplastic anemia, hypothyroidism) | Yes/ No |
| Exposure to drugs* | Administration of medications such as analgesics, antacids, antibacterial drugs, anticoagulants, anticonvulsants, antifungals, antiparasitic drugs, antiretrovirals, antivirals, diuretics, steroids, immunosuppressants, psychotropics, others. | Yes/ No |

*Drug expgosure:

-Analgesics: NSAIDs, paracetamol

-Antacids: omeprazole, ranitidine

-Antibacterial drugs: β-lactams, fluoroquinolones, macrolides, dapsone, ethambutol, daptomycin, linezolid, rifampicin, vancomycin

-Anticoagulants: heparin, low molecular weight heparins (enoxaparin), oral anticoagulants

-Anticonvulsants: valproic acid, carbamazepine, phenytoin, clonazepam

-Antifungals: amphotericin B, flucytosine

-Antiparasitic drugs: pyrimethamine, chloroquine

-Antiretrovirals: abacavir, atazanavir, bictegravir, cobicistat, dolutegravir, efavirenz, elvitegravir, emtricitabine, lamivudine, lopinavir, maraviroc, raltegravir, ritonavir, tenofovir alafenamide, tenofovir disoproxil fumarate, zidovudine

-Antivirals: acyclovir/valacyclovir, ganciclovir/valganciclovir, ribavirin

-Diuretics: spironolactone, furosemide, thiazides

-Steroids: dexamethasone, hydrocortisone, methylprednisolone, prednisone

-Immunosuppressants: methotrexate

-Psychotropics: clozapine, mirtazapine, tricyclic antidepressants (citalopram, escitalopram, fluoxetine, paroxetine, sertraline)

-Others: amiodarone, haloperidol
