# Supplementary material for: Trimethoprim-Sulfamethoxazole-associated early neutropenia in Mexican adults living with HIV: A cohort study
Source: PLoS One. 2023 May 11;18(5):e0285541. doi: 10.1371/journal.pone.0285541 (PMC10174569; doi:10.1371/journal.pone.0285541)
Supplement: S2 Appendix — STROBE Statement—Checklist of items that should be included in reports of cohort studies [1]. (DOCX) [file pone.0285541.s002.docx]

**S3 Appendix. STROBE Checklist Cohort. STROBE Statement—Checklist of items that should be included in reports of cohort studies.[1]**

| Item No. | Section | Recommendation | Page No. | Location in the manuscript |
| --- | --- | --- | --- | --- |
| 1 | **Title and abstract** | (*a*) Indicate the study’s design with a commonly used term in the title or the abstract | 1, 2 | Title & Abstract paragraph 2 |
|  |  | (*b*) Provide in the abstract an informative and balanced summary of what was done and what was found | 2 | Abstract paragraphs 2 and 3 |
|  | Introduction |  |  |  |
| 2 | Background/rationale | Explain the scientific background and rationale for the investigation being reported | 4 | Introduction paragraphs 1 and 2 |
| 3 | Objectives | State specific objectives, including any prespecified hypotheses | 4 | Introduction paragraph 2 |
|  | Methods |  |  |  |
| 4 | Study design | Present key elements of study design early in the paper | 5 | Methods paragraph 1 |
| 5 | Setting | Describe the setting, locations, and relevant dates, including periods of recruitment, exposure, follow-up, and data collection | 5, 6 | Methods paragraphs 1, 3, 4 and 5 |
| 6 | Participants | (*a*) Give the eligibility criteria, and the sources and methods of selection of participants. Describe methods of follow-up | 5, 6 | Methods paragraphs 1, 2 and 5 |
|  |  | (*b*) For matched studies, give matching criteria and number of exposed and unexposed | - | - |
| 7 | Variables | Clearly define all outcomes, exposures, predictors, potential confounders, and effect modifiers. Give diagnostic criteria, if applicable | 6, 7, 8 | Methods paragraphs 4-6, 11, S1 File |
| 8* | Data sources/ measurement | For each variable of interest, give sources of data and details of methods of assessment (measurement). Describe comparability of assessment methods if there is more than one group | 6, 7 | Methods paragraphs 3-5 and 8 |
| 9 | Bias | Describe any efforts to address potential sources of bias | 7 | Methods paragraph 7 |
| 10 | Study size | Explain how the study size was arrived at | 8 | Methods paragraph 13 |
| 11 | Quantitative variables | Explain how quantitative variables were handled in the analyses. If applicable, describe which groupings were chosen and why | 7 | Methods paragraph 8 |
| 12 | Statistical methods | (*a*) Describe all statistical methods, including those used to control for confounding | 8 | Methods paragraphs 9-12. |
|  |  | (*b*) Describe any methods used to examine subgroups and interactions | - | - |
|  |  | (*c*) Explain how missing data were addressed | 5 | Methods paragraph 2 |
|  |  | (*d*) If applicable, explain how loss to follow-up was addressed | 6 | Methods paragraph 5 |
|  |  | (*e*) Describe any sensitivity analyses | - | - |
|  | Results |  |  |  |
| 13* | Participants | (a) Report numbers of individuals at each stage of study—eg numbers potentially eligible, examined for eligibility, confirmed eligible, included in the study, completing follow-up, and analysed | 9 | Fig 1 |
|  |  | (b) Give reasons for non-participation at each stage | 9 | Fig 1 |
|  |  | (c) Consider use of a flow diagram | 9 | Fig 1 |
| 14* | Descriptive data | (a) Give characteristics of study participants (eg demographic, clinical, social) and information on exposures and potential confounders | 9-13 | Results paragraphs 1 and 2, Table 1 and 2 |
|  |  | (b) Indicate number of participants with missing data for each variable of interest | - | - |
|  |  | (c) Summarise follow-up time (eg, average and total amount) | 13 | Results paragraph 3 |
| 15* | Outcome data | Report numbers of outcome events or summary measures over time | 13, 14 | Results paragraph 3, Table 3 |
| 16 | Main results | (*a*) Give unadjusted estimates and, if applicable, confounder-adjusted estimates and their precision (eg, 95% confidence interval). Make clear which confounders were adjusted for and why they were included | 14, 15 | Results paragraph 4, Table 4 |
|  |  | (*b*) Report category boundaries when continuous variables were categorized | - | - |
|  |  | (*c*) If relevant, consider translating estimates of relative risk into absolute risk for a meaningful time period | - | - |
| 17 | Other analyses | Report other analyses done—eg analyses of subgroups and interactions, and sensitivity analyses | - | - |
|  | **Discussion** |  |  |  |
| 18 | Key results | Summarise key results with reference to study objectives | 15 | Discussion paragraph 1 |
| 19 | Limitations | Discuss limitations of the study, taking into account sources of potential bias or imprecision. Discuss both direction and magnitude of any potential bias | 16, 17, 18 | Discussion paragraph 4, 7 |
| 20 | Interpretation | Give a cautious overall interpretation of results considering objectives, limitations, multiplicity of analyses, results from similar studies, and other relevant evidence | 16, 17, 18, 19 | Discussion paragraphs 2, 3, 5, 6, 9, 10 |
| 21 | Generalisability | Discuss the generalisability (external validity) of the study results | 18 | Discussion paragraph 8 |
|  | **Other information** |  |  |  |
| 22 | Funding | Give the source of funding and the role of the funders for the present study and, if applicable, for the original study on which the present article is based | Funding section | Funding paragraph 1 |

*Give information separately for exposed and unexposed groups.

This checklist is copyrighted by the Equator Network. Information on the STROBE Initiative is available at <http://www.strobe-statement.org>.

1. Von Elm E, Altman DG, Egger M, Pocock SJ, Gøtzsche PC, Vandenbroucke JP; STROBE Initiative. The Strengthening the Reporting of Observational Studies in Epidemiology (STROBE) statement: guidelines for reporting observational studies. PLoS Med. 2007 Oct 16;4(10):e296. doi: 10.1371/journal.pmed.0040296. PMID: 17941714; PMCID: PMC2020495.
